# The Effectiveness of Community Action in Reducing Risky Alcohol Consumption and Harm: A Cluster Randomised Controlled Trial

**DOI:** 10.1371/journal.pmed.1001617

**Published:** 2014-03-11

**Authors:** Anthony Shakeshaft, Christopher Doran, Dennis Petrie, Courtney Breen, Alys Havard, Ansari Abudeen, Elissa Harwood, Anton Clifford, Catherine D'Este, Stuart Gilmour, Rob Sanson-Fisher

**Affiliations:** 1 National Drug and Alcohol Research Centre, Faculty of Medicine, UNSW (University of New South Wales), Sydney, New South Wales, Australia; 2 Hunter Medical Research Institute, New Lambton Heights, New South Wales, Australia; 3 Faculty of Health and Medicine, School of Medicine and Public Health, University of Newcastle, Callaghan, New South Wales, Australia; 4 Melbourne School of Population and Global Health, University of Melbourne, Melbourne, Victoria, Australia; 5 Institute for Urban Indigenous Health, Bowen Hills, Queensland, Australia; 6 National Centre for Epidemiology and Population Health, The Australian National University, Canberra, Australia; 7 Department of Global Health Policy, University of Tokyo, Tokyo, Japan; University of Toronto, Canada

## Abstract

**Background:**

The World Health Organization, governments, and communities agree that community action is likely to reduce risky alcohol consumption and harm. Despite this agreement, there is little rigorous evidence that community action is effective: of the six randomised trials of community action published to date, all were US-based and focused on young people (rather than the whole community), and their outcomes were limited to self-report or alcohol purchase attempts. The objective of this study was to conduct the first non-US randomised controlled trial (RCT) of community action to quantify the effectiveness of this approach in reducing risky alcohol consumption and harms measured using both self-report and routinely collected data.

**Methods and Findings:**

We conducted a cluster RCT comprising 20 communities in Australia that had populations of 5,000–20,000, were at least 100 km from an urban centre (population ≥ 100,000), and were not involved in another community alcohol project. Communities were pair-matched, and one member of each pair was randomly allocated to the experimental group. Thirteen interventions were implemented in the experimental communities from 2005 to 2009: community engagement; general practitioner training in alcohol screening and brief intervention (SBI); feedback to key stakeholders; media campaign; workplace policies/practices training; school-based intervention; general practitioner feedback on their prescribing of alcohol medications; community pharmacy-based SBI; web-based SBI; Aboriginal Community Controlled Health Services support for SBI; Good Sports program for sports clubs; identifying and targeting high-risk weekends; and hospital emergency department–based SBI. Primary outcomes based on routinely collected data were alcohol-related crime, traffic crashes, and hospital inpatient admissions. Routinely collected data for the entire study period (2001–2009) were obtained in 2010. Secondary outcomes based on pre- and post-intervention surveys (*n* = 2,977 and 2,255, respectively) were the following: long-term risky drinking, short-term high-risk drinking, short-term risky drinking, weekly consumption, hazardous/harmful alcohol use, and experience of alcohol harm. At the 5% level of statistical significance, there was insufficient evidence to conclude that the interventions were effective in the experimental, relative to control, communities for alcohol-related crime, traffic crashes, and hospital inpatient admissions, and for rates of risky alcohol consumption and hazardous/harmful alcohol use. Although respondents in the experimental communities reported statistically significantly lower average weekly consumption (1.90 fewer standard drinks per week, 95% CI = −3.37 to −0.43, *p* = 0.01) and less alcohol-related verbal abuse (odds ratio = 0.58, 95% CI = 0.35 to 0.96, *p* = 0.04) post-intervention, the low survey response rates (40% and 24% for the pre- and post-intervention surveys, respectively) require conservative interpretation. The main limitations of this study are as follows: (1) that the study may have been under-powered to detect differences in routinely collected data outcomes as statistically significant, and (2) the low survey response rates.

**Conclusions:**

This RCT provides little evidence that community action significantly reduces risky alcohol consumption and alcohol-related harms, other than potential reductions in self-reported average weekly consumption and experience of alcohol-related verbal abuse. Complementary legislative action may be required to more effectively reduce alcohol harms.

**Trial registration:**

Australian New Zealand Clinical Trials Registry ACTRN12607000123448

*Please see later in the article for the Editors' Summary*

## Abstract 2

In a cluster randomized controlled trial, Anthony Shakeshaft and colleagues measure the effectiveness of a multi-component community-based intervention for reducing alcohol-related harm.

## Editors' Summary

**Background:**

People have consumed alcoholic beverages throughout history, but alcohol use is now an increasing global public health problem. According to the World Health Organization's 2010 Global Burden of Disease Study, alcohol use is the fifth leading risk factor (after high blood pressure and smoking) for disease and is responsible for 3.9% of the global disease burden. Alcohol use contributes to heart disease, liver disease, depression, some cancers, and many other health conditions. Alcohol also affects the well-being and health of people around those who drink, through alcohol-related crimes and road traffic crashes. The impact of alcohol use on disease and injury depends on the amount of alcohol consumed and the pattern of drinking. Most guidelines define long-term risky drinking as more than four drinks per day on average for men or more than two drinks per day for women (a “drink” is, roughly speaking, a can of beer or a small glass of wine), and short-term risky drinking (also called binge drinking) as seven or more drinks on a single occasion for men or five or more drinks on a single occasion for women. However, recent changes to the Australian guidelines acknowledge that a lower level of alcohol consumption is considered risky (with lifetime risky drinking defined as more than two drinks a day and binge drinking defined as more than four drinks on one occasion).

**Why Was This Study Done?:**

In 2010, the World Health Assembly endorsed a global strategy to reduce the harmful use of alcohol. This strategy emphasizes the importance of community action–a process in which a community defines its own needs and determines the actions that are required to meet these needs. Although community action is highly acceptable to community members, few studies have looked at the effectiveness of community action in reducing risky alcohol consumption and alcohol-related harm. Here, the researchers undertake a cluster randomized controlled trial (the Alcohol Action in Rural Communities [AARC] project) to quantify the effectiveness of community action in reducing risky alcohol consumption and harms in rural communities in Australia. A cluster randomized trial compares outcomes in clusters of people (here, communities) who receive alternative interventions assigned through the play of chance.

**What Did the Researchers Do and Find?:**

The researchers pair-matched 20 rural Australian communities according to the proportion of their population that was Aboriginal (rates of alcohol-related harm are disproportionately higher among Aboriginal individuals than among non-Aboriginal individuals in Australia; they are also higher among young people and males, but the proportions of these two groups across communities was comparable). They randomly assigned one member of each pair to the experimental group and implemented 13 interventions in these communities by negotiating with key individuals in each community to define and implement each intervention. Examples of interventions included general practitioner training in screening for alcohol use disorders and in implementing a brief intervention, and a school-based interactive session designed to reduce alcohol harm among young people. The researchers quantified the effectiveness of the interventions using routinely collected data on alcohol-related crime and road traffic crashes, and on hospital inpatient admissions for alcohol dependence or abuse (which were expected to increase in the experimental group if the intervention was effective because of more people seeking or being referred for treatment). They also examined drinking habits and experiences of alcohol-related harm, such as verbal abuse, among community members using pre- and post-intervention surveys. After implementation of the interventions, the rates of alcohol-related crime, road traffic crashes, and hospital admissions, and of risky and hazardous/harmful alcohol consumption (measured using a validated tool called the Alcohol Use Disorders Identification Test) were not statistically significantly different in the experimental and control communities (a difference in outcomes that is not statistically significantly different can occur by chance). However, the reported average weekly consumption of alcohol was 20% lower in the experimental communities after the intervention than in the control communities (equivalent to 1.9 fewer standard drinks per week per respondent) and there was less alcohol-related verbal abuse post-intervention in the experimental communities than in the control communities.

**What Do These Findings Mean?:**

These findings provide little evidence that community action reduced risky alcohol consumption and alcohol-related harms in rural Australian communities. Although there was some evidence of significant reductions in self-reported weekly alcohol consumption and in experiences of alcohol-related verbal abuse, these findings must be interpreted cautiously because they are based on surveys with very low response rates. A larger or differently designed study might provide statistically significant evidence for the effectiveness of community action in reducing risky alcohol consumption. However, given their findings, the researchers suggest that legislative approaches that are beyond the control of individual communities, such as alcohol taxation and restrictions on alcohol availability, may be required to effectively reduce alcohol harms. In other words, community action alone may not be the most effective way to reduce alcohol-related harm.

**Additional Information:**

Please access these websites via the online version of this summary at http://dx.doi.org/10.1371/journal.pmed.1001617.

- The World Health Organization provides detailed information about alcohol; its fact sheet on alcohol includes information about the global strategy to reduce the harmful use of alcohol; the Global Information System on Alcohol and Health provides further information about alcohol, including information on control policies around the world
- The US National Institute on Alcohol Abuse and Alcoholism has information about alcohol and its effects on health
- The US Centers for Disease Control and Prevention has a website on alcohol and public health that includes information on the health risks of excessive drinking
- The UK National Health Service Choices website provides detailed information about drinking and alcohol, including information on the risks of drinking too much, tools for calculating alcohol consumption, and personal stories about alcohol use problems
- MedlinePlus provides links to many other resources on alcohol
- More information about the Alcohol Action in Rural Communities project is available

## Introduction

Alcohol use contributed an estimated 3.9% to the global burden of disease in 2010, moving from the eighth highest ranked risk factor in 1990 to the fifth highest ranked risk factor in 2010 [1]. The World Health Organization advocates community action to reduce risky alcohol consumption and harm, arguing that all members of a community are responsible for action because the burden of alcohol harm is spread across multiple settings, including health services, police services, public spaces, and workplaces [2]. Community action is also highly acceptable to members of communities [3]. Nevertheless, the results of only six randomised trials of the effectiveness of alcohol community action have been published [4]–[9], all of which were US-based, focused on young people (the unit of randomisation was schools in three trials [4]–[6] and university campuses in two trials [8],[9], rather than the community), and limited to self-report or alcohol purchase attempt outcomes. There is no rigorous evidence about whether the economic benefits of alcohol community action outweigh its costs.

The Alcohol Action in Rural Communities (AARC) project was a cluster randomised controlled trial (RCT) aimed at quantifying the effectiveness of community action in reducing risky alcohol consumption and harm, including the first benefit–cost analysis undertaken in any country to estimate the economic impact of community action on alcohol harms. The pre-specified hypotheses were that, post-intervention, the experimental communities, relative to the control communities, would have lower proportions of survey respondents reporting long-term risky drinking, short-term high-risk drinking, short-term risky drinking, and alcohol-related harms; fewer alcohol-related crime and traffic incidents; and a short-term increase in alcohol-related hospital admissions for alcohol dependence or abuse as more people sought, or were referred to, treatment.

## Methods

### Ethics and Trial Registration

The research was approved by the Human Research Ethics Committee of the University of Newcastle (the administering institution), and all participants provided informed consent. The trial was registered with the Australian New Zealand Clinical Trials Registry (registration number ACTRN12607000123448). Outcome data were analysed by Dr. Dennis Petrie, Stuart Gilmour, and Ansari Abudeen under the direction of Professor Catherine D'Este and Professor Anthony Shakeshaft. The benefit–cost analysis was performed by Dr. Dennis Petrie and Ansari Abudeen under the direction of Professor Christopher Doran. The trial was not registered prospectively because it commenced earlier than the 1 July 2005 date agreed by the International Committee of Medical Journal Editors for compulsory trial registration (the trial was retrospectively submitted for registration on 20 November 2006 and formally registered on 12 February 2007). Detailed methods, including descriptions of the interventions, are provided elsewhere [10],[11].

### Study Design and Community Selection

The study design was a cluster RCT with communities as the unit of randomisation. A cluster RCT is the most appropriate design to assess the community-level impact of a community-action intervention. Communities in New South Wales (NSW), Australia, were invited to participate if they had a population between 5,000 and 20,000 (*n* = 27) [12], were at least 100 km away from an urban centre (population ≥ 100,000) (*n* = 24), and were not involved in another alcohol-related community project (*n* = 20). Communities with these characteristics are large enough to have sufficient resources to implement multiple interventions while allowing detailed observation of the impact of alcohol harms and the effectiveness of interventions in different settings (e.g., the effect of improved management of alcohol dependence by primary care physicians on demand for inpatient hospital beds) [13],[14]. Although limiting the AARC project to rural communities allowed rates of risky alcohol consumption and harms in different communities to be examined, and facilitated detailed observation of the effectiveness of the intervention [3],[15]–[22], the community-action approach and the use of both routinely collected and survey data are readily applicable to urban communities [23].

### Randomisation

Proportions of males, young people, and Aboriginal individuals across communities were potential matching variables, given their disproportionately high rates of alcohol-related harm [24]–[26]. Since the proportions of males and the proportions of people aged 15–24 y were similar across communities, communities were ranked, in decreasing order, according to the proportion of their population defined as Aboriginal. Contiguous communities in the list were provisionally classified as matched pairs. Each pair was checked to ensure the communities were at least 100 km apart geographically, to minimise the risk of cross-contamination of intervention impacts. One community in each pair was randomly allocated to the experimental condition using an Excel program. It was not possible to blind the ten experimental communities to their condition. The mayor of each experimental community consented to the involvement of his or her community in the project, and facilitated the implementation of the interventions in his or her community. The author with statistical expertise (C. D'E.) supervised the randomisation procedure, and the authors responsible for the implementation of the interventions (A. S. and C. D.) were blinded to the randomisation process.

### Intervention Descriptions

The 13 selected interventions and the timing of their implementation in the experimental communities are identified in Table 1. They are described in detail elsewhere [10],[11] and summarised as follows.

**Table 1:** Summary of the interventions and the timeline of their implementation, and the timing of the community surveys.

Intervention	Intervention Period						2010
	Pre-Intervention 2001–2004	Intervention Initiation 2005	Post-Intervention				
			2006	2007	2008	2009	
1. Community engagement^a^	×^b^	×					
Pre-intervention community survey		×					
2. GP training in alcohol SBI c ^,^ d	×^e^	×					
3. Feedback to key stakeholders		×	×	×	×	×	
4. Media campaign		×	×	×	×	×	
5. Workplace policies/practices training a ^,^ f		×					
6. School-based intervention f		×	×				
7. GP feedback on their prescribing of alcohol medications a			×				
8. Community pharmacy-based SBI^c^			×	×			
9. Web-based SBI^c^			×	×			
10. Aboriginal Community Controlled Health Services support for SBI^c^				×	×	×	
11. Good Sports program for sports clubs d ^,^ g				×	×	×	
12. Identifying and targeting high-risk weekends				×	×	×	
13. Hospital ED-based SBI c						×	
Post-intervention community survey							×

a This intervention was expected to have an ongoing impact over the post-intervention period (2006–2009).

b Commenced March 2004.

c No GP SBI training occurred in control communities.

d The timing of these interventions was dictated by opportunities to expand existing programs to include the AARC experimental communities.

e Commenced October 2004.

f The timing of these interventions was dictated by having access to the expertise needed to develop and implement the interventions relatively quickly.

g Eight of the control communities also had at least one sports club enrolled in this program.

#### Community engagement

The process of inviting communities to participate in the AARC project, and obtaining their commitment to help design and implement the interventions, required both direct and indirect engagement. Direct engagement involved working with community stakeholders (such as the mayor and police) to engage and promote the view that alcohol-related harm was a community-wide issue that required a community-wide response. Regular meetings were held to clarify the project and identify roles. Indirect engagement occurred with government departments that had administrative oversight for staff based in the communities, to obtain support for their involvement.

#### General practitioner training in alcohol screening and brief intervention

As part of a broader general practitioner (GP) training program being implemented concurrently in NSW, clinical addiction specialists provided two 2-h training sessions for local GPs in screening and brief intervention (SBI), using the ten-item Alcohol Use Disorders Identification Test (AUDIT) with a standard drink chart [27]. Feedback was based on the FLAGS process, used as part of the adopted Drinkless Kit, comprising the following: feedback to patients on their level of drinking relative to normative data, listening to patients' views on their own drinking patterns and behaviours, advising patients on lower risk levels of drinking and the benefits they would obtain from drinking less, goal setting, and identifying strategies to help achieve goals. None of these GP training sessions were held in an AARC control community.

#### Feedback to key stakeholders

During the engagement process, the communities nominated a group of key stakeholders who became a community coalition group with whom the researchers liaised as the project progressed. The coalition was responsible for assisting in implementing the locally agreed interventions and ensuring that data feedback was appropriate.

#### Media campaign

A media campaign coincided with every new or updated data analysis and with the implementation and completion of interventions. The media campaign was restricted to local newspapers and radio to help prevent contamination of the control communities.

#### Workplace policies/practices training

All major employers in each community were identified and offered a choice of workplace interventions of different levels of intensity that best met their need. The simplest level comprised mailed information about the project and appropriate alcohol-related workplace policies and procedures, followed by a phone call to ensure the information had been received and to clarify any issues. For interested workplaces, the second level of intervention involved the provision of a resources kit in the mail. The third option was to participate in a face-to-face, 6-h training workshop with representatives from other major employers in their community.

#### School-based intervention

Year 11 students (16- to 17-y-olds) were provided with a 1-h interactive session carefully targeted at preventing alcohol harm among young people. Year 12 students were excluded because of their final year high-school and study commitments. The session was developed and presented by the Media Manager of Australia's National Drug and Alcohol Research Centre.

#### GP feedback on their prescribing of alcohol medications

A letter was sent to each GP in the experimental communities to attempt to increase the frequency with which they prescribe an appropriate pharmacotherapy to their alcohol-dependent patients [14]. The letter provided information, specifically tailored to their community, on the likely number of alcohol-dependent individuals (estimated from data collected in the AARC pre-intervention survey) and current rates of prescribing of these medications, and a summary of the current evidence on their effectiveness.

#### Community pharmacy-based SBI

Pharmacists were provided with a coloured page comprising the ten-item AUDIT, with instructions for completion and scoring on the front and feedback for each level of risk on the back. These were made available on counters in pharmacies or placed in bags with other purchases.

#### Web-based SBI

This intervention also used the ten-item AUDIT, providing immediate personalised feedback to respondents on screen. This intervention was made available from January 2006 and was advertised widely when launched, but its use was very low, and so it was stopped in 2008.

#### Aboriginal Community Controlled Health Services support for SBI

Three communities had indigenous-specific medical services, generically called Aboriginal Community Controlled Health Services. These Aboriginal Community Controlled Health Services agreed to undergo alcohol SBI training similar to that provided to GPs and to trial a process of integrating SBI into their current information technology systems, to examine whether this would assist clinicians and health workers in providing SBI routinely [28].

#### Good Sports program for sporting clubs

When the AARC project commenced, the Australian Drug Foundation had begun to implement a program to reduce alcohol-related harm in sporting clubs across NSW, called the Good Sports program. Since six of the ten intervention communities were involved in Good Sports, the AARC project provided funding to ensure that the additional four were also included in the Good Sports program. Eight of the control communities had at least one sports club enrolled in the program.

#### Identifying and targeting high-risk weekends

In each AARC experimental community, the research team used alcohol-related crime data from the previous 7 y to identify those weekends with disproportionately high rates of alcohol-related crime. A total of 115 high-risk weekends were identified for the experimental communities, meaning an average of four high-risk weekends per year were targeted in each of the ten experimental communities over the three years 2007–2009. Those weekends were targeted with the co-ordinated implementation of multiple strategies: the mayor wrote to venues licensed to sell alcohol in the week leading up to the problematic weekend to encourage them to be vigilant about their responsible service of alcohol requirements; there was a media campaign; local police agreed to increase their visibility by conducting foot or car patrols late at night and early in the morning on the Friday and Saturday of the problematic weekend, especially around licensed venues and the central business district; and there was feedback immediately after the targeted weekend from the research team to the local media on the number of alcohol-related crimes that had occurred, compared to the same weekends in previous years [29]. For the control communities, 116 problematic weekends were likewise identified, but no intervention was implemented.

#### Hospital emergency department–based SBI

The five AARC communities that had emergency departments (EDs) with electronic records agreed to provide screening and mail brief intervention to all patients presenting to the ED for treatment during a 10-month period in 2009. As with the pharmacist- and GP-based SBI, this screening used the AUDIT questionnaire to screen all patients who presented to a participating ED. Personalised feedback was subsequently mailed to participants by the research team, providing them with information about their level of drinking, relative to other people in their community, and with advice on low-risk levels of alcohol consumption [30].

### Intervention Selection, Implementation, and Costs

These 13 interventions were selected by identifying existing research evidence, obtaining the views of communities and alcohol professionals about the types of interventions they thought were important, and then negotiating with a key stakeholder group in each community to specifically define and implement each intervention [3],[21]. GP training in SBI and a Good Sports program were exceptions, both of which were implemented in all communities opportunistically to coincide with other projects. A unique feature of this study was that it co-ordinated different types of interventions (e.g., SBI, GP prescribing, media campaign) implemented in different settings (e.g., primary care, schools, workplaces). Commencement of the intervention phase was planned for 2005. Although a lag period was anticipated between the implementation of the interventions and their effectiveness, the duration of this lag for different types of interventions was unknown. Consequently, 2001–2004 was defined as pre-intervention, 2005 as intervention initiation, and 2006–2009 as post-intervention. As summarised in Table 2, the total cost of designing and implementing the AARC interventions was estimated at $608,102 in 2006 Australian dollars.

**Table 2:** Summary of the AARC project interventions and their costs.

Intervention	Resource Value (in 2006 Australian Dollars)
1. Community engagement	55,517
2. GP training in alcohol SBI	26,167
3. Feedback to key stakeholders	81,718
4. Media campaign	195,393
5. Workplace policies/practices training	27,655
6. School-based intervention	13,098
7. GP feedback on their prescribing of alcohol medications	10,482
8. Community pharmacy-based SBI	2,959
9. Web-based SBI	3,593
10. Aboriginal Community Controlled Health Services support for SBI	22,908
11. Good Sports program for sports clubs	66,000
12. Identifying and targeting high-risk weekends	78,462
13. Hospital ED-based SBI	24,151
**Total**	**608,102**

### Measures

#### Community characteristics

Characteristics available for all 20 communities and likely to be associated with rates of alcohol consumption and harm were obtained. Australian Bureau of Statistics data were used to identify the proportions of males, young people (aged 15–24 y), and Aboriginal Australians [12] in each community, their Accessibility/Remoteness Index of Australia score for 1999 [31], and their Socio-Economic Index for Areas score [32]. The number of premises licensed to sell alcohol was obtained, separately for pubs/clubs, wholesalers/retailers, and others (e.g., restaurants) [33], as were numbers of full-time police/highway patrol officers (from NSW Police) and general practitioners (from Divisions of General Practice).

#### Routinely collected data

De-identified unit record data for all 20 communities were obtained for crime, road traffic crashes, and hospital inpatient admissions. Crime data were obtained from the NSW Bureau of Crime Statistics and Research, traffic crash data from the NSW Roads and Traffic Authority, and hospital inpatient admission data from the NSW Ministry of Health (the original study protocol included ED data as an outcome, but these were excluded because only five communities had data available electronically). To ensure uniformity, minimise zero counts, and control for seasonal variation, all data for the entire study period (2001–2009) were obtained in 2010 and analysed by quarter (36 data points per community).

#### Survey data

Pre- and post-intervention surveys were designed to identify community-level proportions of long-term risky drinking, short-term risky drinking, short-term high-risk drinking, hazardous/harmful drinking, experience of alcohol-related verbal abuse, and average weekly alcohol consumption, none of which are captured in routinely collected datasets [11]. Individuals in all 20 communities were eligible for the survey if they were aged between 18 and 62 y, reflecting the Australian legal age for voting at the lower end and the likely low contribution to community-level alcohol harms at the upper end [34]. Survey participants were randomly selected in sex and 5-y age strata from the Australian Electoral Roll, accessed through the Australian Electoral Commission for the pre-intervention survey and the NSW Electoral Commission for the post-intervention survey (i.e., the same roll was accessed through two different commissions due to a change in access rules). The Australian Electoral Roll comprises an estimated 95% of Australian residents aged at least 18 y (voting is compulsory in Australia). Both surveys were designed to be completed within 15 min and were able to be understood by an average 13- to 15-y-old (Flesch reading score of 65). Items included demographic characteristics, experiences of alcohol-related harm (adapted from major Australian surveys [35],[36]), and personal alcohol use (the ten-item AUDIT with a standard drink chart [27]).

#### Outcomes

Outcome measures comprised routinely collected data (primary outcomes) and survey data (secondary outcomes). For routinely collected crime data, proxy measures were developed and tested to identify those that were alcohol-related because the reliability and validity with which alcohol-related crimes are routinely identified is unknown [19],[37]. Alcohol-related crimes were identified by adapting a NSW-level proxy measure to the AARC communities, comprising street offences (offensive conduct, offensive language, and wilful and obscene exposure), assaults (actual bodily harm, grievous bodily harm, and common assault), malicious damage to property, and an aggregate of these (total crime) that occurred at times that are typically alcohol-related: Sunday 10 pm–Monday 6 am, Monday 10 pm–Tuesday 2 am, Wednesday 10 pm–Thursday 2 am, Friday 10 pm–Saturday 6 am, and Saturday 6 pm–Sunday 6 am [37].

Alcohol-related traffic crashes were identified using an AARC-specific proxy measure defined as any traffic crash that occurred in the following alcohol-related times: Friday 10 pm–Saturday 6 am, Saturday 6 pm–Sunday 10 am, and Sunday 6 pm–Sunday 10 pm [25].

Alcohol-related inpatient hospital admissions were defined as those with an International Classification of Diseases (ICD-10) principal diagnosis code of F10.2 (alcohol dependence) or F10.0 or F10.1 (alcohol abuse). Only the principal diagnosis code was used because the reliability and validity of the secondary codes is unknown [38],[39], and only these two conditions (alcohol dependence and alcohol abuse) were included because they are wholly attributable to alcohol [40].

Survey data outcomes were as follows: (1) long-term risky drinking, defined by the then current Australian National Health and Medical Research Council's drinking guidelines as the consumption of more than 28 (men) or 14 (women) standard drinks per week [41]; (2) short-term risky drinking (more than six [males] or four [females] standard drinks on one occasion [41]); (3) short-term high-risk drinking (more than ten [males] or six [females] standard drinks on one occasion [41]); (4) hazardous/harmful drinking (total AUDIT score ≥ eight [42]); (5) experienced at least one incident of verbal abuse in the past 12 mo by someone affected by alcohol; and (6) average weekly consumption, calculated by multiplying each respondent's answers to the first two AUDIT questions [43]. Routinely collected data outcomes that were alcohol-related were as follows: (1) total crime, (2) assaults, (3) malicious damage, (4) street offences, (5) total traffic crashes, (6) persons injured in a traffic crash, (7) number of crashes with no injury/fatality, (8) inpatient hospital admissions for alcohol dependence, and (9) inpatient hospital admissions for alcohol abuse.

### Statistical Methods

All data analyses used Stata/MP 11.2 and a two-sided 5% significance level. Unmatched analysis was used because it is more powerful than matched analysis when the number of communities is small and the correlation between the matching variable and the outcome is low (≤0.2) [44]. Negative binominal models were estimated because the count outcomes were over-dispersed (crime, road traffic crashes, and hospital inpatient admissions). Population size was used as the exposure variable. Logistic regression models were estimated for binary survey outcomes (risky drinking and verbal abuse), and a linear regression model was estimated for the continuous survey outcome (average weekly consumption).

All models were fitted using generalised estimating equations with an exchangeable correlation structure to adjust for clustering of individuals within communities. The models included terms for intervention group (experimental or control), the survey period (pre- or post-intervention), and their interaction. The interaction term assessed whether the post-intervention difference between the experimental and control groups was statistically significantly different to the pre-intervention difference. For the logistic and linear models, sex and age group were included as covariates given their known association with alcohol harm [24]–[26]. For count data, three terms were included for seasonal variation, and an additional term for year and quarter (to adjust for trends over time). In addition to these adjusted models, which account for possible sources of variation other than the intervention, results are also presented for an unadjusted analysis, which compares the experimental and control communities in the post-intervention period, controlling only for clustering (and population size for the count data). The standard errors of parameters in the generalised estimating equation models were estimated using the jack-knife method given that the number of clusters was small (*n*<50) [45].

For the logit and negative binomial models, the interaction terms are not relative risks (RRs) or odds ratios (ORs) per se, but rather are the post-intervention ratios divided by the pre-intervention ratios: a result less than one indicates positive intervention effectiveness. Alternatively, these can be considered as the relative ratio (risk or odds) for experimental versus control communities post-intervention, adjusted for the pre-intervention ratio. For the linear regression model, the interaction term is the post-intervention difference in means between intervention and control groups, minus the pre-intervention difference.

### Sample Sizes

For routinely collected data (primary outcomes), the availability of ten experimental and ten control communities allowed detection of relative between-group differences in outcomes of 30% (80% power, *p*≤0.05), assuming a pre-intervention outcome rate of 10 per 1,000 population and a coefficient of variation of 0.1. For survey data (secondary outcomes), a sample size of 1,200 respondents per group was required to detect absolute differences on self-reported outcomes of 8 percentage points (dichotomous variables) and 0.16 of a standard deviation (continuous variables) as statistically significant, given ten communities per group and assuming a design effect of 2 (80% power, *p*≤0.05). Since a response rate of 40%–50% was anticipated, based on national surveys [35], a sample of 8,000 was required (400/community). Given that the pre-intervention survey response rate was at the lower end of the anticipated range, an additional 2,000 respondents were randomly selected for the post-intervention survey. To control for response rate bias (females and older people were over-represented) [15], survey data were weighted to reflect the age and gender characteristics of each community. Weights were calculated as the proportion of the population in each age and gender stratum, divided by the proportion of the survey respondents in each stratum.

## Results

### Community Characteristics

Table 3 shows that the experimental and control communities were comparable pre-intervention across all community characteristics.

**Table 3:** Community-level summary statistics pre-intervention, separately for experimental and control communities.

Community Characteristic	Experimental Communities (*n* = 10)	Control Communities (*n* = 10)
**Percent young males (15–24 y)**	6.1 (0.5)	5.9 (0.4)
**Percent Aboriginal or Torres Strait Islander**	4.9 (2.6)	4.9 (4.5)
**ARIA score** a	2.9 (0.6)	2.9 (2.0)
**SEIFA score** b	958 (23)	956 (26)
**Number of premises licensed to sell alcohol** c	28 (7.7)	27 (7.1)
Number of hotels/clubs licensed to sell alcohol^c^	11 (4.0)	10 (3.8)
Number of alcohol wholesalers/retailers^c^	4 (2.4)	3 (1.7)
Number of other premises licensed to sell alcohol^c^	13 (5.0)	14 (5.6)
**Number of full-time police** c	14 (4.7)	21 (12)
**Number of full-time highway patrollers** c	4 (1.1)	3.4 (2.3)
**Number of GPs** c	9.6 (3.8)	12 (7.8)

Data are mean (standard deviation).

a Accessibility/Remoteness Index of Australia score–higher scores indicate greater remoteness.

b Socio-Economic Index for Areas score–higher scores indicate greater advantage.

c Per 10,000 population.

### Survey Response Rates and Sample Characteristics

Of the 7,985 potential participants mailed a pre-intervention survey (the actual number mailed to each community ranged from 394 to 401 because of rounding in age and sex categories), 405 were no longer at the supplied address and 3,017 of the remaining 7,580 responded (40%). Of the 9,984 potential participants mailed a post-intervention survey, 455 were no longer at the supplied address and 2,278 of the remaining 9,529 responded (24%). Table 4 compares the demographic characteristics of survey respondents for the pre- and post-intervention surveys, both within and between the experimental and control communities. The only statistically significant differences were that a greater proportion of respondents for the post-intervention survey, in both the experimental and control communities, reported a gross household weekly income of at least AUD$700, compared to respondents for the pre-intervention survey. This nominal increase in household income most likely reflects inflation between 2005 and 2010.

**Table 4:** Demographic characteristics of the experimental and control communities for the pre-intervention (2005) and post-intervention (2010) surveys.

Characteristic	Pre-Intervention Survey *n* = 2,977	Post-Intervention Survey *n* = 2,255
**Age in years (mean)**		
Experimental	40.0 (39.4–40.6)	41.3 (40.4–42.3)
Control	40.3 (39.7–40.9)	41.7 (40.8–42.5)
**Gender (percent male)**		
Experimental	49.7 (46.6–52.8)	50.0 (46.3–53.6)
Control	50.8 (47.8–53.8)	41.7 (40.8–42.5)
**Aboriginal or Torres Strait Islander (percent)**		
Experimental	2.6 (1.6–3.6)	2.5 (1.3–3.7)
Control	2.0 (0.9–3.1)	2.7 (1.1–4.2)
**Unemployed (percent)**		
Experimental	2.4 (1.4–3.4)	2.1 (1.1–3.1)
Control	2.4 (1.5–3.3)	3.3 (1.9–4.6)
**Post-school qualification (percent)**		
Experimental	53.9 (50.8–56.9)	54.0 (50.3–57.7)
Control	51.9 (48.8–54.8)	52.0 (48.2–55.8)
**Married or de facto** c **(percent)**		
Experimental	68.8 (65.9–71.6)	70.9 (67.4–74.4)
Control	69.0 (66.1–71.8)	65.8 (62.3–69.3)
**Income ≥ AUD$700 (percent)** a		
Experimental	55.5 (52.5–58.6)	72.1 (68.9–75.2)^b^
Control	59.4 (56.5–62.4)	66.9 (63.2–70.5)^b^

Data are mean (95% CI) or percent (95% CI).

a Gross weekly household income ≥ AUD$700 per week.

b Statistically significant difference–the CI for the post-intervention survey does not overlap with the CI for the pre-intervention survey.

c For the purpose of the survey, de facto means living with a partner without a formal marriage/civil union.

### Intervention Outcomes

#### Routinely collected data (primary outcomes)

**Figure 1:**
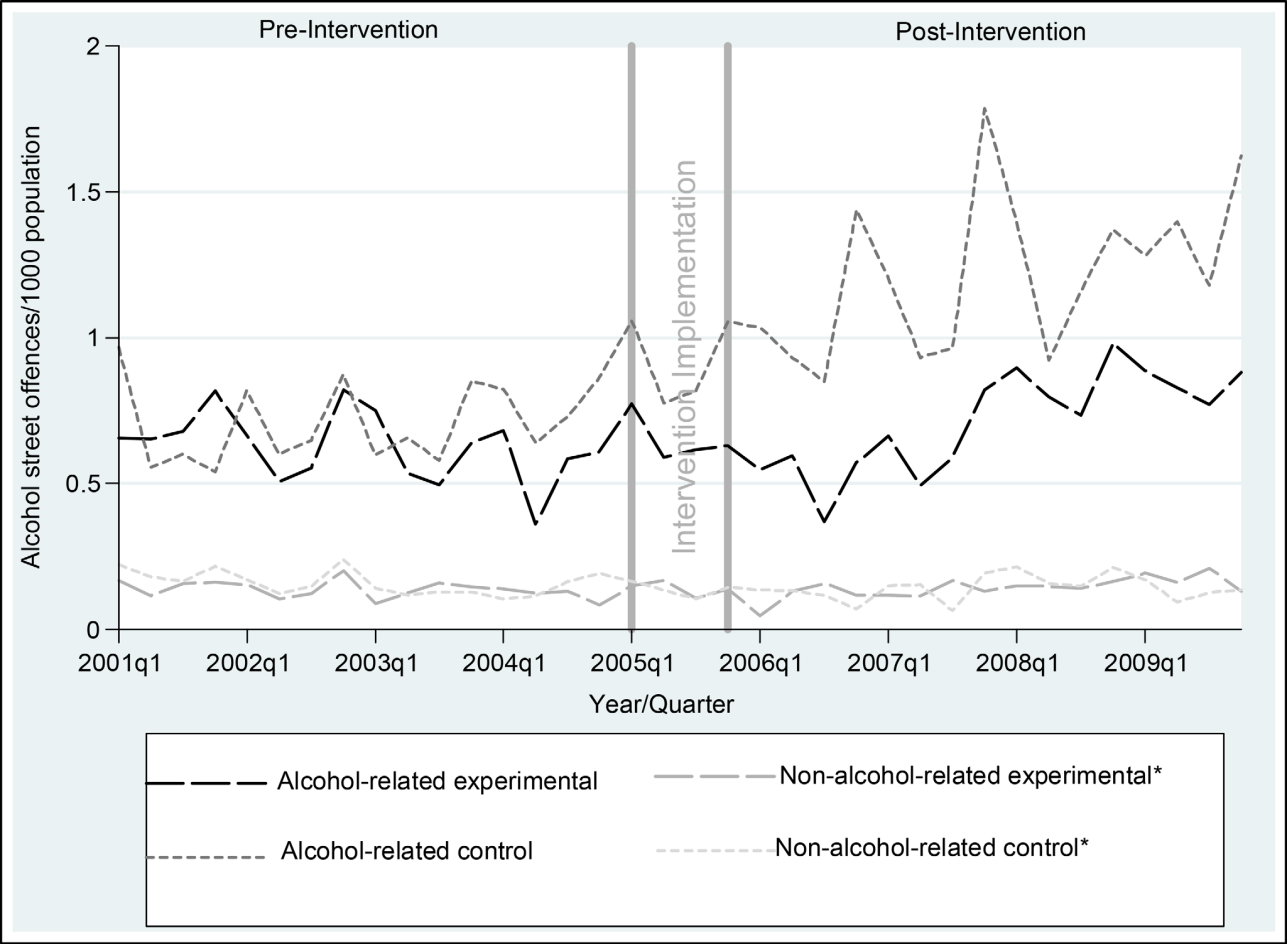
Rates of alcohol-related street offences per 1,000 population, per quarter, for experimental and control communities, 1 January 2001–31 December 2009. *Non-alcohol-related times were graphed to check whether the intervention simply shifted crimes from alcohol to non-alcohol times. Non-alcohol times were Monday 6 am–Monday 6 pm, Tuesday 6 am–Tuesday 2 pm, Wednesday 10 am–Wednesday 2 pm, Thursday 6 am–Thursday 2 pm, and Friday 6 am–Friday 10 am [37].

**Figure 2:**
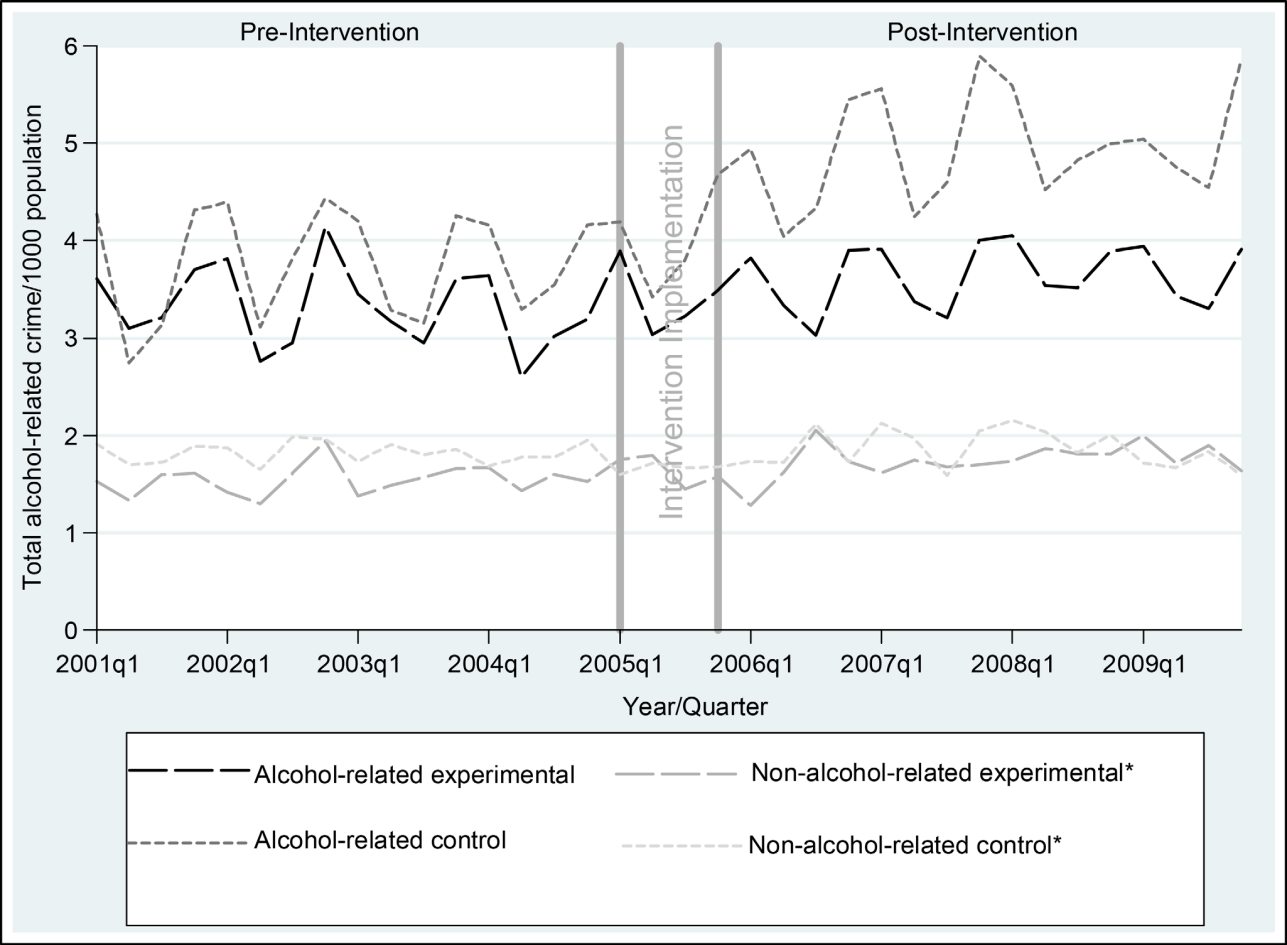
Rates of total alcohol-related crime per 1,000 population, per quarter, for experimental and control communities, 1 January 2001–31 December 2009. *Non-alcohol-related times were graphed to check whether the intervention simply shifted crimes from alcohol to non-alcohol times. Non-alcohol times were Monday 6 am–Monday 6 pm, Tuesday 6 am–Tuesday 2 pm, Wednesday 10 am–Wednesday 2 pm, Thursday 6 am–Thursday 2 pm, and Friday 6 am–Friday 10 am [37].

The seasonal variation in alcohol-related street offences evident in Figure 1 (generally higher in summer) was replicated for assaults, malicious damage, and total crime, and was statistically significant for all crime outcomes (Table S1). There were no statistically significant seasonal effects for alcohol-related traffic crashes or inpatient hospital admissions for alcohol dependence or abuse, except for more alcohol-related traffic crashes that did not result in an injury or fatality in the October–December quarter (Table S1). In the pre-intervention period, alcohol-related crime was similar for the experimental and control communities, the number of alcohol-related traffic crashes in the experimental communities was about half that in the control communities, and the number of inpatient admissions for both alcohol dependence and abuse was lower in the experimental communities, although the only statistically significant difference was fewer inpatient admissions for alcohol dependence in the experimental communities (RR = 0.50, 95% CI = 0.29–0.86, *p* = 0.02) (Table 5). Post-intervention, after adjusting for pre-intervention values, there were no statistically significant differences in favour of the experimental communities. The unadjusted analysis showed statistically significantly fewer inpatient admissions for alcohol dependence in the experimental communities post-intervention (RR = 0.50, 95% CI = 0.25–1.00, *p* = 0.05). In Figure 1, alcohol-related street offences are graphed as rate per 1,000 population to delineate the pattern of results over time for the most significant of the alcohol-related crime outcomes. Figure 1 shows a widening gap between the experimental and control communities in the post- relative to the pre-intervention period, with no evidence of a shift in street offences from alcohol- to non-alcohol-related times, and no evidence of an absolute reduction in alcohol-related street offences. This pattern was replicated for total alcohol-related crimes (Figure 2).

**Table 5:** Effectiveness of the intervention on alcohol-related crime, traffic crashes, and inpatient hospital admissions.

Outcome	Pre- or Post-Intervention^a^	Crude Prevalence^b^				Post-Intervention Differences				
		Experimental Communities		Control Communities		Adjustment	RR	95% CI	*t*-Test^c^	*p*-Value
		*n*	Rate	*n*	Rate					
**Alcohol-related crime**										
Total alcohol-related crime	Pre	174	13.2	191	15.1					
	Post	196	14.5	237	19.8	Adjusted^d^	0.83	0.66–1.05	−1.68	0.11
						Unadjusted^e^	0.74	0.48–1.13	−1.49	0.15
Alcohol-related assaults	Pre	61	4.3	75	5.6					
	Post	68	4.8	90	7.2	Adjusted^d^	0.86	0.66–1.13	−1.15	0.26
						Unadjusted^e^	0.67	0.42–1.07	−1.80	0.09
Alcohol-related malicious damage	Pre	81	5.9	81	6.0					
	Post	90	6.5	92	7.2	Adjusted^d^	0.91	0.73–1.13	−0.90	0.38
						Unadjusted^e^	0.89	0.65–1.22	−0.77	0.45
Alcohol-related street offences	Pre	32	2.5	35	2.8					
	Post	38	2.9	55	4.9	Adjusted^d^	0.67	0.44–1.02	−2.00	0.06
						Unadjusted^e^	0.59	0.26–1.33	−1.36	0.19
**Alcohol-related traffic crashes**										
Total number of alcohol-related crashes	Pre	10	0.80	20	1.55					
	Post	10	0.73	18	1.42	Adjusted^d^	1.00	0.74–1.36	−0.01	1.00
						Unadjusted^e^	0.78	0.26–2.31	−0.48	0.64
Number of persons injured	Pre	6.2	0.49	11	0.85					
	Post	6.0	0.43	10	0.81	Adjusted^d^	0.96	0.57–1.61	−0.17	0.87
						Unadjusted^e^	0.76	0.30–1.93	−0.63	0.54
Number of crashes with no injury/fatality	Pre	5.9	0.44	11	0.89					
	Post	5.5	0.41	11	0.87	Adjusted^d^	0.93	0.71–1.22	−0.55	0.59
						Unadjusted^e^	0.75	0.24–2.30	−0.55	0.59
**Alcohol-related inpatient hospital admissions**										
Inpatient admissions for alcohol dependence	Pre^f^^,^ g	6.1	0.43	12	0.80					
	Post	8.2	0.56	14	1.06	Adjusted^d^	1.00	0.48–2.08	−0.01	0.99
						Unadjusted^e^	0.50	0.25–1.00	−2.10	0.05^g^
Inpatient admissions for alcohol abuse	Pre	8.8	0.63	12	0.82					
	Post	19	1.28	16	1.09	Adjusted^d^	1.58	0.98–2.53	2.01	0.06
						Unadjusted^e^	1.14	0.64–2.04	0.47	0.64

a 2005 data were excluded from this analysis to allow for the anticipated lag between the implementation and effectiveness of the intervention.

b Number (*n*) and rate per 1,000 population (rate) per year, averaged across experimental (*n* = 10) and control (*n* = 10) communities.

c Standard error obtained using jack-knife estimate because of the small number of clusters.

d Adjusted for over-dispersion, correlation within communities, community population size, seasonal effects, trends over time, and pre-intervention values. The interaction term tests whether the post-intervention RR for experimental communities is statistically significantly different to that of control communities, after adjusting for pre-intervention ratios.

e Unadjusted analysis, controlling for clustering (and population size) only.

f Statistically significantly fewer admissions pre-intervention in the experimental, compared to control, communities (RR = 0.50, 95% CI = 0.29 to 0.86, *p* = 0.02).

g Statistically significant at the 5% level.

#### Self-reported data (secondary outcomes)

In the pre-intervention period, self-reported alcohol consumption and harms were similar for experimental and control communities (Table 6). Post-intervention, after adjusting for pre-intervention values, there were no statistically significant differences in favour of the experimental communities, except for lower average weekly alcohol consumption (1.90 fewer standard drinks per week, 95% CI = −3.37 to −0.43, *p* = 0.01) and fewer experiences of alcohol-related verbal abuse (OR = 0.58, 95% CI = 0.35 to 0.96, *p* = 0.04). This pattern of results was replicated in the unadjusted analysis.

**Table 6:** Effectiveness of the intervention on self-reported consumption and harms.

Outcome	Pre- or Post-Intervention	Crude Prevalence^a^				Post-Intervention Differences				
		Experimental Communities		Control Communities		Adjustment	OR^d^	95% CI	*z*-Test^e^	*p*-Value
		*n* b	Percent^c^	*n* b	Percent^c^					
**Logistic regression outcomes**										
Long-term risky drinking	Pre	130	9.2	129	8.7					
	Post	101	9.3	131	12	Adjusted^f^	0.69	0.46–1.04	−1.76	0.08
						Unadjusted^g^	0.69	0.46–1.04	−1.79	0.07
Short-term risky drinking (past 12 mo)	Pre	609	43	629	42					
	Post	507	47	512	49	Adjusted^f^	0.96	0.65–1.42	−0.19	0.90
						Unadjusted^g^	0.93	0.64–1.35	−0.39	0.70
Short-term high-risk drinking (past 12 mo)	Pre	286	20	265	18					
	Post	214	20	248	23	Adjusted^f^	0.69	0.45–1.07	−1.67	0.09
						Unadjusted^g^	0.70	0.46–1.06	−1.69	0.09
Hazardous/harmful drinking (AUDIT score ≥ 8)	Pre	351	24	333	22					
	Post	243	21	269	24	Adjusted^f^	0.80	0.55–1.18	−1.14	0.30
						Unadjusted^g^	0.76	0.53–1.10	−1.44	0.10
										
Experience of alcohol-related verbal abuse	Pre	289	20	270	18					
	Post	158	14	226	20	Adjusted^f^	0.58	0.35–0.96	−2.11	0.04^h^
						Unadjusted^g^	0.56	0.34–0.92	−2.31	0.02^h^
**Linear regression outcome**										
Average consumption (standard drinks per week)	Pre	8.1	0.3	7.5	0.3					
	Post	7.8	0.4	9.5	0.6	Adjusted^i^	−1.90	−3.37 to −0.43	−2.53	0.01^h^
						Unadjusted^g^	−2.28	−3.96 to −0.60	−2.66	0.01^h^

a Average number of respondents at-risk in each community (n) and the proportion averaged across experimental (*n* = 10) and control (*n* = 10) communities.

b Mean for linear regression outcome.

c Standard error for linear regression outcome.

d Mean difference for linear regression outcome.

e Standard error obtained using jack-knife estimate because of the small number of clusters.

f Adjusted for age and sex distributions, and pre-intervention values. The interaction term tests whether the post-intervention OR for experimental communities is statistically significantly different to that of control communities, after adjusting for pre-intervention ratios.

g Unadjusted analysis, controlling for clustering only.

h Statistically significant at the 5% level.

i Adjusted for age and sex distributions, and pre-intervention values. The interaction term tests whether the post-intervention mean for experimental communities is statistically significantly different to control communities, after adjusting for pre-intervention means.

## Discussion

### Summary of Findings

At the 5% level of statistical significance, there was insufficient evidence from the routinely collected data (primary outcomes) to conclude that the intervention was effective in the experimental, relative to control, communities for alcohol-related crime, traffic crashes, or hospital inpatient admissions, or rates of risky alcohol consumption or hazardous/harmful alcohol use, as measured by AUDIT. Although the unadjusted analysis showed statistically significantly fewer inpatient admissions for alcohol dependence in the experimental communities post-intervention, relative to the control communities, this result most likely reflects that pre-intervention inpatient admissions for alcohol dependence were also statistically significantly lower in the experimental communities (Table 5): the post-intervention difference was not statistically significant after adjusting for this pre-intervention difference. Figure 1 shows that alcohol-related street offences, as the most significant of the alcohol-related crime outcomes, did not reduce over time, but did increase at a slower rate in the experimental, relative to the control, communities. Based on the survey data (secondary outcomes), respondents in the experimental communities post-intervention reported statistically significantly lower average weekly consumption and less alcohol-related verbal abuse.

### Methodological Considerations and Implications

The results that were not statistically significant are unlikely to be a consequence of unobserved bias: an RCT design was used, and pre-intervention community characteristics likely to be associated with rates of alcohol consumption and harm were comparable in the experimental and control communities (Table 3). Bias related to the statistical methods is also unlikely: the jack-knife method was used to control for possible under-estimation of the variance of parameters in the generalised estimating equation models; adjustment for multiple comparisons was not required given that the majority of findings showed no intervention effectiveness; and the results that were not statistically significant showed small to moderate effectiveness and high levels of uncertainty (wide 95% CIs). This last observation may reflect that the study had insufficient power to detect any true intervention effectiveness as statistically significant because of the limited sample size of 20 communities, or that there was variation in the extent to which communities were exposed to the different interventions, or that the observed differences were smaller than those hypothesised. The web-based intervention, for example, had low rates of use across all communities, while the community engagement, feedback to stakeholders, media campaign, and school-based interventions were applied consistently because the researchers had a relatively high degree of control over their implementation. Conversely, although researchers provided the relevant training and resources, the extent to which GPs, pharmacists, EDs, workplaces, sports clubs, and police (on high-risk weekends) actually implemented their respective interventions in practice most likely varied substantially between communities.

Interpreting the statistically significant results is also difficult. Although the survey response rates were low (40% and 24% for the pre- and post-intervention periods, respectively), they were weighted to control for response rate bias, the survey items had at least some evidence for their reliability and validity (e.g., AUDIT [27]), there was consistency within the self-report outcomes (e.g., a significant reduction in average weekly consumption is consistent with a reduction in the proportion of individuals reporting long-term risky drinking), and there was consistency between self-report and routinely collected data (e.g., a significant reduction in verbal abuse is consistent with a marginally significant reduction in street offences, while a lack of effectiveness on self-reported short-term risky drinking is consistent with a lack of effectiveness on alcohol-related assaults). If the impact on average weekly alcohol consumption truly represents the effectiveness of the intervention, it equates to a 20% reduction in average alcohol consumption in the experimental communities (1.9 fewer standard drinks per week [intervention effectiveness]/9.5 standard drinks per week [mean post-intervention consumption in the control communities]). This could provide a practical health benefit to the individuals who are consuming less alcohol and to communities: a 10% reduction in average consumption in a community has been estimated to equate to 25% fewer individuals engaging in heavy drinking (≥24 standard drinks/week) [46].

### Conclusions

To our knowledge, this study provides the most methodologically rigorous data to date of the impact of community action, as defined by the suite of interventions implemented, on risky alcohol consumption and harm. It provides insufficient evidence to conclude that community action is effective in reducing a range of risky alcohol consumption patterns and alcohol-related harms, other than potentially reducing average weekly consumption and rates of alcohol-related verbal abuse. Despite these modest outcomes, the broader AARC project provides some unique insights for community-action approaches.

First, unlike existing randomised trials of alcohol community action [4]–[9], the AARC project showed that it is possible to use routinely collected data to measure the impact of community-based interventions, and to identify rates of different types of alcohol-related harm in different communities [19],[47], although the data may need to be tailored to measure a specific outcome (e.g. measuring alcohol-related serious assaults only) and to the psychometric properties of the specific measure examined [25],[37]. This study demonstrates the potential for key stakeholders to identify and target the particular harms that are most problematic in their own communities.

Second, although the AARC project is the first randomised trial of community action to focus on alcohol outcomes across the whole community, as opposed to only among young people, its results were broadly comparable to those of the existing randomised trials of alcohol community action: one found no impact on primary outcomes [5], while the remainder found some impacts on self-reported outcomes [4],[6]–[9]. Given that the only statistically significant effects in the AARC project were also on self-reported (secondary) outcomes rather than routinely collected data (primary) outcomes, it might be that community action is more effective on the less severe harms and drinking behaviours that are reported by respondents than on the more severe harms that are recorded in routinely collected datasets. Alternatively, these findings may highlight a potential limitation of self-reported data associated with a range of methodological factors, such as their reliability, their validity, and survey response rates.

Third, the AARC project showed that the majority of survey respondents agree with the idea of a community-action approach [3], and that households are willing to pay to reduce alcohol-related harm [16]. Indeed, the economic analysis of the AARC project used a counterfactual methodology to estimate a cost–benefit ratio that was favourable, essentially because of the value households placed on reducing alcohol-related harm [11].

Fourth, the primary purpose of this RCT was to test the collective impact of a suite of interventions that were feasible for communities themselves to implement and enforce. The intervention costs (Table 2), for example, highlight that communities could implement all 13 interventions for approximately AUD$61,000 (total cost of AUD$608,102 divided by ten experimental communities). Despite the interventions being feasible for communities to implement, the modest outcomes suggest that legislative approaches that are beyond the direct control of communities, such as pricing mechanisms [48], restrictions on alcohol availability [19],[48],[49], and drink driving laws targeting young people [47],[50], may be more cost-effective than community action in actually reducing a wide range of alcohol-related harms, as opposed to limiting the rate of increase of a subset of harms. Indeed, the general increase in alcohol-related crime over time in both the experimental and control communities (Figures 1 and 2) may reflect the current strength of alcohol legislation in Australia. This interpretation challenges the view that community action alone is most likely to sustainably reduce alcohol-related harm.

## Supporting Information

Checklist S1 **CONSORT 2010 checklist of information to include when reporting a cluster randomised trial.**
(PDF)

Table S1 **Seasonal variation in alcohol-related harms.**
(DOCX)

Text S1 **CONSORT flow diagram for a community-level cluster randomised controlled trial: the Alcohol Action in Rural Communities project.**
(PDF)

## Abbreviations

AARC: Alcohol Action in Rural Communities
AUDIT: Alcohol Use Disorders Identification Test
ED: emergency department
GP: general practitioner
NSW: New South Wales
OR: odds ratio
RCT: randomised controlled trial
RR: relative risk
SBI: screening and brief intervention
