# Supplementary material for: The Effectiveness of Community Action in Reducing Risky Alcohol Consumption and Harm: A Cluster Randomised Controlled Trial
Source: PLoS Med. 2014 Mar 11;11(3):e1001617. doi: 10.1371/journal.pmed.1001617 (PMC3949675; doi:10.1371/journal.pmed.1001617)
Supplement: Checklist S1 — CONSORT 2010 checklist of information to include when reporting a cluster randomised trial. (PDF) [file pmed.1001617.s001.pdf]

Table 1: CONSORT 2010 checklist of information to include when reporting a cluster randomised trial

Section/Topic	Item No	Standard Checklist item	Extension for cluster designs	Page No *
**Title and abstract**				
	1a	Identification as a randomised trial in the title	Identification as a cluster randomised trial in the title	Title of the manuscript.
	1b	Structured summary of trial design, methods, results, and conclusions (for specific guidance see CONSORT for abstracts)[1,2]	See table 2	Abstract of the manuscript.
**Introduction**				
**Background and objectives**	2a	Scientific background and explanation of rationale	Rationale for using a cluster design	Paragraph 1 of the introduction.<br><br>Paragraph under sub-heading “Study design and community selection.”
	2b	Specific objectives or hypotheses	Whether objectives pertain to the the cluster level, the individual participant level or both	Paragraph 2 of the introduction.
**Methods**				
**Trial design**	3a	Description of trial design (such as parallel, factorial) including allocation ratio	Definition of cluster and description of how the design features apply to the clusters	Paragraph under sub-heading “Study design and community selection.”
	3b	Important changes to methods after trial commencement (such as eligibility criteria), with reasons		Change in access to electoral roll data – paragraph under sub-heading “survey data.”<br><br>Change to an unmatched analysis – 1st paragraph under the sub-heading “Statistical methods.”
**Participants**	4a	Eligibility criteria for participants	Eligibility criteria for clusters	Eligibility for clusters (communities) – paragraph under sub-heading “Study design and community

				selection."<br><br>Eligibility for participants in the survey data - paragraph under sub-heading "survey data."
	4b	Settings and locations where the data were collected		Description of the size of the clusters - paragraph under sub-heading "Study design and community selection."<br><br>Description of the characteristics of the clusters – Table 3.<br><br>Descriptions of the data sources – first 3 paragraphs under sub-heading "Measures."
**Interventions**	5	The interventions for each group with sufficient details to allow replication, including how and when they were actually administered	Whether interventions pertain to the cluster level, the individual participant level or both	Descriptions of the interventions, how they were selected and the order of their implementation – under the sub-heading "intervention descriptions" and Tables 1 and 2.
**Outcomes**	6a	Completely defined pre-specified primary and secondary outcome measures, including how and when they were assessed	Whether outcome measures pertain to the cluster level, the individual participant level or both	Described under the sub-heading "Outcomes."
	6b	Any changes to trial outcomes after the trial commenced, with reasons		N/A – no changes
**Sample size**	7a	How sample size was determined	Method of calculation, number of clusters(s) (and whether equal or unequal cluster sizes are assumed), cluster size, a coefficient of intracluster correlation (ICC or *k*), and an indication of its uncertainty	Described under the sub-heading "Sample sizes."
	7b	When applicable, explanation of any interim analyses and stopping guidelines		N/A
**Randomisation:**				

**Sequence generation**	8a	Method used to generate the random allocation sequence		Described under the sub-heading “Randomisation.”
	8b	Type of randomisation; details of any restriction (such as blocking and block size)	Details of stratification or matching if used	Described under the sub-heading “Randomisation.”
**Allocation concealment mechanism**	9	Mechanism used to implement the random allocation sequence (such as sequentially numbered containers), describing any steps taken to conceal the sequence until interventions were assigned	Specification that allocation was based on clusters rather than individuals and whether allocation concealment (if any) was at the cluster level, the individual participant level or both	Described under the sub-heading “Randomisation.”
**Implementation**	10	Who generated the random allocation sequence, who enrolled participants, and who assigned participants to interventions	Replace by 10a, 10b and 10c	N/A for a cluster trial
	10a		Who generated the random allocation sequence, who enrolled clusters, and who assigned clusters to interventions	Described under the sub-heading “Randomisation.”
	10b		Mechanism by which individual participants were included in clusters for the purposes of the trial (such as complete enumeration, random sampling)	Complete enumeration applies to the interventions and routinely collected outcome data (described under syb-headings “Intervention selection, implementation and costs” and “Routinely collected data.” Random sampling applies to the survey outcome data (described under the sub-heading “Survey data.”
	10c		From whom consent was sought (representatives of the cluster, or individual cluster members, or both), and whether consent was sought before or after randomisation	Described under the sub-heading “Randomisation.”

**Blinding**	11a	If done, who was blinded after assignment to interventions (for example, participants, care providers, those assessing outcomes) and how		Described under the sub-heading "Randomisation."
	11b	If relevant, description of the similarity of interventions		N/A
**Statistical methods**	12a	Statistical methods used to compare groups for primary and secondary outcomes	How clustering was taken into account	Described in the 2nd paragraph under the sub-heading "Statistical methods."
	12b	Methods for additional analyses, such as subgroup analyses and adjusted analyses		Described under the sub-heading "Statistical methods."
**Results**				
**Participant flow (a diagram is strongly recommended)**	13a	For each group, the numbers of participants who were randomly assigned, received intended treatment, and were analysed for the primary outcome	For each group, the numbers of clusters that were randomly assigned, received intended treatment, and were analysed for the primary outcome	A CONSORT flow diagram is attached as an uploaded document. All communities randomly assigned received all interventions and were included in all analyses.
	13b	For each group, losses and exclusions after randomisation, together with reasons	For each group, losses and exclusions for both clusters and individual cluster members	There were no losses at the community (cluster level). Response rates and respondent characteristics for the survey data are described in the paragraph under the sub-heading "Survey response rates and sample characteristics."
**Recruitment**	14a	Dates defining the periods of recruitment and follow-up		Described in Table 1.
	14b	Why the trial ended or was stopped		N/A
**Baseline data**	15	A table showing baseline demographic and clinical characteristics for each	Baseline characteristics for the individual and cluster levels as applicable for each group	Tables 3 and 4.

		group		
**Numbers analysed**	16	For each group, number of participants (denominator) included in each analysis and whether the analysis was by original assigned groups	For each group, number of clusters included in each analysis	At the cluster level, data from all 20 communities were analysed. At the individual level, the number of surveys analysed is described in the paragraph under the sub-heading "Survey response rates and sample characteristics."
**Outcomes and estimation**	17a	For each primary and secondary outcome, results for each group, and the estimated effect size and its precision (such as 95% confidence interval)	Results at the individual or cluster level as applicable and a coefficient of intracluster correlation (ICC or k) for each primary outcome	Primary outcomes reported under sub-heading "Routinely collected data (primary outcomes)" and Table 5.<br>Secondary outcomes reported under sub-heading "Self-reported data (secondary outcomes)" and Table 6.
	17b	For binary outcomes, presentation of both absolute and relative effect sizes is recommended		See Table 6.
**Ancillary analyses**	18	Results of any other analyses performed, including subgroup analyses and adjusted analyses, distinguishing pre-specified from exploratory		Both adjusted and unadjusted analyses are presented in Tables 5 and 6. Descriptions of the unadjusted analysis are in the 2nd paragraph under the subheading "Statistical methods."
**Harms**	19	All important harms or unintended effects in each group (for specific guidance see CONSORT for harms[3])		N/A
**Discussion**				
**Limitations**	20	Trial limitations, addressing sources of potential bias, imprecision, and, if relevant, multiplicity of analyses		A description of methodological issues and limitations is provided under the sub-heading "Methodological considerations and implications."
**Generalisability**	21	Generalisability (external validity, applicability) of the trial findings	Generalisability to clusters and/or individual participants (as relevant)	Last sentence of the paragraph under sub-heading "Study design and community

				selection.”
**Interpretation**	22	Interpretation consistent with results, balancing benefits and harms, and considering other relevant evidence		Interpretation provided under the sub-heading “Conclusions.”
**Other information**				
**Registration**	23	Registration number and name of trial registry		Provided under the sub-heading “Ethics and trial registration.”
**Protocol**	24	Where the full trial protocol can be accessed, if available		References 10 and 11, and in the trial registry documentation.
**Funding**	25	Sources of funding and other support (such as supply of drugs), role of funders		The project was funded by the Foundation for Alcohol Research and Education, an independent charitable organisation (http://www.fare.org.au/about-us/). The Australian Government provides core funding to the National Drug and Alcohol Research Centre through the Substance Misuse Prevention and Service Improvement Grants Fund.

** Note: page numbers optional depending on journal requirements*

---

1 Hopewell S, Clarke M, Moher D, Wager E, Middleton P, Altman DG, et al. CONSORT for reporting randomised trials in journal and conference abstracts. *Lancet* 2008, 371:281-283

2 Hopewell S, Clarke M, Moher D, Wager E, Middleton P, Altman DG at al (2008) CONSORT for reporting randomized controlled trials in journal and conference abstracts: explanation and elaboration. *PLoS Med* 5(1): e20

3 Ioannidis JP, Evans SJ, Gotzsche PC, O'Neill RT, Altman DG, Schulz K, Moher D. Better reporting of harms in randomized trials: an extension of the CONSORT statement. *Ann Intern Med* 2004; 141(10):781-788.
