# Supplementary material for: The Effectiveness of Community Action in Reducing Risky Alcohol Consumption and Harm: A Cluster Randomised Controlled Trial
Source: PLoS Med. 2014 Mar 11;11(3):e1001617. doi: 10.1371/journal.pmed.1001617 (PMC3949675; doi:10.1371/journal.pmed.1001617)
Supplement: Table S1 — Seasonal variation in alcohol-related harms. (DOCX) [file pmed.1001617.s002.docx]

**Table S1: Seasonal variation in alcohol-related harms**

| **Outcomes^a^** | **Incidence-Rate Ratio** | **Standard error** | **95% CI** | |
| --- | --- | --- | --- | --- |
|  |  |  | **Lower** | **Upper** |
| **Total alcohol-related crime** |  |  |  |  |
| April-June | 0.81* | 0.02 | 0.77 | 0.85 |
| July-September | 0.83* | 0.01 | 0.80 | 0.86 |
| October-December | 1.02 | 0.04 | 0.94 | 1.10 |
|  |  |  |  |  |
| **Alcohol-related assaults** |  |  |  |  |
| April-June | 0.78* | 0.02 | 0.74 | 0.83 |
| July-September | 0.77* | 0.02 | 0.73 | 0.82 |
| October-December | 0.96 | 0.03 | 0.90 | 1.03 |
|  |  |  |  |  |
| **Alcohol-related malicious damage** |  |  |  |  |
| April-June | 0.86* | 0.04 | 0.79 | 0.94 |
| July-September | 0.94 | 0.04 | 0.86 | 1.02 |
| October-December | 1.07 | 0.05 | 0.98 | 1.17 |
|  |  |  |  |  |
| **Alcohol-related street offences** |  |  |  |  |
| April-June | 0.81* | 0.04 | 0.73 | 0.90 |
| July-September | 0.82* | 0.04 | 0.74 | 0.91 |
| October-December | 1.09 | 0.09 | 0.92 | 1.29 |
| **Total alcohol-related crashes** |  |  |  |  |
| April-June | 1.01 | 0.07 | 0.89 | 1.16 |
| July-September | 1.08 | 0.08 | 0.93 | 1.26 |
| October-December | 1.09 | 0.07 | 0.95 | 1.25 |
|  |  |  |  |  |
| **Alcohol-related crashes with injured persons** |  |  |  |  |
| April-June | 1.01 | 0.14 | 0.75 | 1.34 |
| July-September | 1.06 | 0.13 | 0.82 | 1.37 |
| October-December | 1.00 | 0.10 | 0.81 | 1.23 |
|  |  |  |  |  |
| **Alcohol-related crashes with no injury/fatality** |  |  |  |  |
| April-June | 1.02 | 0.10 | 0.84 | 1.25 |
| July-September | 1.08 | 0.00 | 0.89 | 1.30 |
| October-December | 1.12* | 0.06 | 1.00 | 1.26 |
| **Inpatient admissions for alcohol dependence** |  |  |  |  |
| April-June | 0.84 | 0.10 | 0.65 | 1.07 |
| July-September | 0.86 | 0.09 | 0.70 | 1.07 |
| October-December | 0.89 | 0.09 | 0.71 | 1.11 |
|  |  |  |  |  |
| **Inpatient admissions for alcohol abuse** |  |  |  |  |
| April-June | 0.87 | 0.08 | 0.72 | 1.04 |
| July-September | 0.90 | 0.10 | 0.72 | 1.13 |
| October-December | 0.97 | 0.08 | 0.81 | 1.15 |

*^a^Alcohol-related outcomes (each quarter relative to January-March), 2001-2009.*

**Significant at the 5% level – indicated where the 95% Confidence Interval (95%CI) excludes 1.*
