# Supplementary material for: The Effectiveness of Community Action in Reducing Risky Alcohol Consumption and Harm: A Cluster Randomised Controlled Trial
Source: PLoS Med. 2014 Mar 11;11(3):e1001617. doi: 10.1371/journal.pmed.1001617 (PMC3949675; doi:10.1371/journal.pmed.1001617)
Supplement: Text S1 — CONSORT flow diagram for a community-level cluster randomised controlled trial: the Alcohol Action in Rural Communities project. (PDF) [file pmed.1001617.s003.pdf]

CONSORT FLOW DIAGRAM FOR A COMMUNITY-LEVEL CLUSTER RCT:
THE ALCOHOL ACTION IN RURAL COMMUNITIES (AARC) PROJECT*

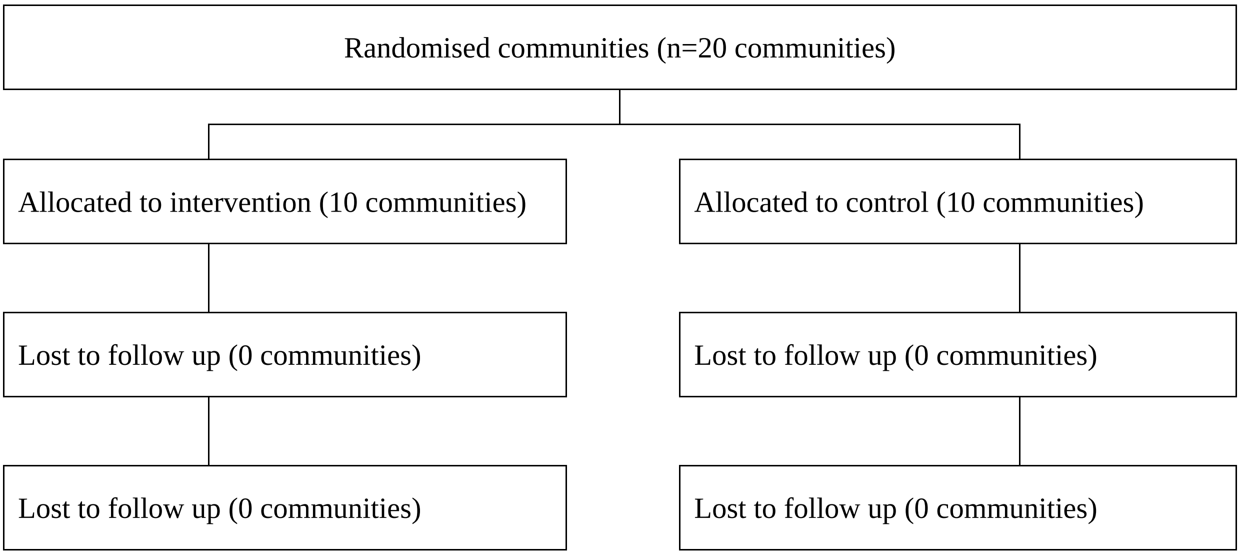

*Note: this flow diagram is based on the Figure 4 example of the *"Consort 2010 statement: extension to cluster randomised trials."* Nevertheless, it is different to a standard cluster RCT where the intervention and the statistical analysis are designed for individuals within randomly selected clusters. An innovation of this trial is that the intervention and the statistical analysis are designed for the whole community (ie: the impact of the interventions is assessed at the community-level, not on individuals within communities or clusters)
